# C-Peptide Inhibits Decidualization in Human Endometrial Stromal Cells via GSK3β-PP1

**DOI:** 10.3389/fcell.2020.609551

**Published:** 2020-11-30

**Authors:** Sana Abdul Khaliq, Mi-Ock Baek, Hye-Jeong Cho, Seung Joo Chon, Mee-Sup Yoon

**Affiliations:** ^1^Department of Molecular Medicine, School of Medicine, Gachon University, Incheon, South Korea; ^2^Lee Gil Ya Cancer and Diabetes Institute, Gachon University, Incheon, South Korea; ^3^Department of Health Sciences and Technology, GAIHST, Gachon University, Incheon, South Korea; ^4^Department of Obstetrics and Gynecology, Gachon University Gil Medical Center, College of Medicine, Gachon University, Incheon, South Korea

**Keywords:** C-peptide, GSK3 β, PP1, decidualization, senescence, apoptosis

## Abstract

Decidualization refers to the functional differentiation of endometrial stromal cells and plays a significant role in embryo implantation and pregnancy. C-peptide is excreted in equimolar concentrations as that of insulin during the metabolism of proinsulin in pancreatic beta-cells. High levels of C-peptide are correlated with hyperinsulinemia and polycystic ovarian syndrome, which show a defect in decidualization. However, the role of C-peptide in decidualization has not yet been studied. Here, we identified C-peptide as an endogenous antideciduogenic factor. This inhibitory function was confirmed by the reduced expression of decidual markers, including prolactin, insulin-like growth factor-binding protein-1, and Forkhead box protein O1 as well as by the fibroblastic morphological change in the presence of C-peptide. C-peptide also enhanced cellular senescence and decreased the proportion of apoptotic cells during decidualization. In addition, C-peptide potentiated the inhibitory effects of both insulin and palmitic acid in an AKT- and autophagy-independent manner, respectively. Furthermore, C-peptide augmented protein phosphatase 1 (PP1) activity, leading to a reduction in the inhibitory phosphorylation of glycogen synthase kinase (GSK)3β, which resulted in enhanced cellular senescence and decreased apoptosis during decidualization. Taken together, our findings suggest that C-peptide is an antideciduogenic factor acting via the regulation between PP1 and GSK3β in patients with hyperinsulinemia.

## Introduction

The human proinsulin connecting peptide (C-peptide) is composed of 31 amino acids, which are cleaved from proinsulin during proteolytic processing (Bhatt et al., 2014; Leno-Duran et al., 2014). C-peptide links and stabilizes the A- and B- chains of insulin molecules, resulting in its proper folding and interchain disulfide bond formation. C-peptide and insulin are secreted in equimolar concentrations from the pancreatic β-cells (Beischer et al., 1976; Wahren et al., 2000), with C-peptide largely viewed as an inert marker for insulin production with negligible metabolism in the liver (Hills and Brunskill, 2009). However, recent studies have revealed several important physiological and protective functions of C-peptide in diabetic complications, suggesting that this byproduct may play a hormone-like role in diverse aspects of cellular signaling.

It has been suggested that C-peptide exerts its effect by binding to Gαi-coupled G-protein-coupled receptor (GPCR) on the cellular surface, which is supported by a previous study that demonstrated high-affinity interactions between a fluorophore-labeled C-peptide and human cell membranes (Rigler et al., 1999). C-peptide has been reported to activate phospholipase C (PLC) – Ca^2+^-dependent signaling pathways, thereby increasing the activity of both Na^+^-K^+^-ATPase and endothelial nitric oxide synthase. It has also been shown to stimulate mitogen-activated protein kinase (MAPK), leading to the activation of Na^+^-K^+^-ATPase and numerous transcription factors, including cAMP response element-binding protein (CREB), nuclear factor kappa beta (NF-κB), and activating transcription factor 1 (ATF1). These functions may play some protective roles in diabetic vascular dysfunction, micro- and/or macro-vascular damage, and diabetic neuropathy (Wahren et al., 2000). In addition, C-peptide induces cyclin D1 expression, promoting the critical transition of cell proliferation from G1- to S-phase via the activation of retinoblastoma protein phosphorylation in smooth muscles of both rats and humans (Walcher et al., 2006). These results suggest that C-peptide plays several functional roles in diverse tissues and biological contexts, in addition to its role in diabetes. However, the potential effects of C-peptide signaling on endometrium differentiation during embryo implantation and pregnancy maintenance have not yet been investigated.

Decidualization is the process of transformation of endometrial fibroblasts to secretory round-shaped decidual cells during the menstrual cycle and pregnancy (Gellersen and Brosens, 2014). Elevated progesterone levels during the mid-secretory phase of the menstrual cycle induce a decidua-like morphological change in the stromal cells that surround the spiral arteries in the endometrium (Okada et al., 2018). During this process, the decidual stromal cells provide the key nutrients to support placental development and ultimately embryo implantation (Dunn et al., 2003). The extended cytoplasm of the decidual stromal cells contains glycogen and lipid droplets and an increased number of intracellular phagosomes and lysosomes, possibly contributing to the intense remodeling of the extracellular matrix (ECM) and the release of diverse cytokines and signaling molecules (Gellersen and Brosens, 2014). In addition, these cells express diverse signal coordinators, which serve as decidual markers, including prolactin (PRL), insulin-like growth factor-binding protein-1 (IGFBP1), Forkhead box protein O1 (FOXO1), and the NODAL-signaling pathway inhibitor left-right determination factor 2 (LEFTY2).

A high level of C-peptide is closely accompanied by hyperinsulinemia and insulin resistance (Hills and Brunskill, 2009), which have been linked to several endocrine conditions and pregnancy complications. Patients with polycystic ovarian syndrome (PCOS), characterized by hyperandrogenism and hyperinsulinemia, have a high risk of recurrent pregnancy loss and miscarriage (Chakraborty et al., 2013). Treatment of patients with PCOS with metformin reduces the occurrence of early pregnancy loss (Nawaz and Rizvi, 2010), suggesting that insulin resistance or hyperinsulinemia may play a significant role in early pregnancy loss in PCOS patients. Hence, we hypothesize that C-peptide under hyperinsulinemia affects decidualization and subsequently maintaining pregnancy. Here, we examined for the first time whether C-peptide played a role in decidualization and further investigated the mechanistic regulation involved. Our findings reveal a novel role for C-peptide in the endometrium and provide an explanation for the high risk of recurrent pregnancy loss in patients with hyperinsulinemia.

## Materials and Methods

### Antibodies and Other Reagents

The primary antibodies used in this study are listed in Supplementary Table 1. The secondary antibodies were obtained from Jackson Immuno Research Laboratories Inc. (West Grove, PA, United States) (anti-mouse #115-035-003; anti-rabbit# 211-002-171). The C-peptide was synthesized by Peptrone (Daejon, South Korea). A palmitic acid (PA) solution was prepared using 20 mM PA in 150 mM NaCl with 5% bovine serum albumin. Okadaic acid (OA) was dissolved in DMSO. A list of the reagents used in this study is provided in Supplementary Table 2.

### Isolation and Culture of Human eSCs

Human endometrial stromal cells (human eSCs) were isolated from the human endometrium acquired through hysterectomies of 25 premenopausal women aged 46–52 years. The participants showed no sign of glucose metabolism irregularities, diabetes, or PCOS and underwent surgery for non-endometrial abnormalities at the Gil Hospital. The information of participants is presented in Supplementary Table 3. All experiments were performed in compliance with the relevant guidelines and regulations of Gachon University (GAIRB2018-301). Written informed consent was obtained from all participants. Isolation of eSCs was performed following a previously reported procedure (Yoon et al., 2007). After isolation, the cells were used under 4 passages. Human eSCs were grown in Dulbecco’s modified Eagle’s medium (DMEM) containing 1 g/L glucose and supplemented with 10% fetal bovine serum (FBS) at 37°C with 5% CO_2_ and were detached from the plate using 0.05% trypsin-EDTA (Welgene, Gyeongsangbuk-do, South Korea). *In vitro* decidualization was induced by plating the cells and growing them to 100% confluency by treating them with DMEM containing 10% FBS, 1% penicillin/streptomycin (10,000 U/mL), and 0.5 mM 8-bromo adenosine 3′5′-cyclic adenosine monophosphate (8-Br-cAMP). Fresh medium was provided on alternate days (Baek et al., 2018).

### Cell Lysis and Western Blot Analysis

Human eSCs were washed once with ice-cold phosphate-buffered saline (PBS), scraped, and lysed with 1X lysis buffer (Cell Signaling Technology). The supernatant was collected after microcentrifugation at 13,000 × *g* for 10 min and then boiled for 5 min in sodium dodecyl sulfate (SDS) sample buffer (Baek et al., 2019). Proteins were then subjected to SDS-polyacrylamide gel electrophoresis and transferred to polyvinylidene fluoride membranes (Millipore, Billerica, MA, United States). These membranes were blocked with 5% skimmed milk phosphate buffered saline with 0.1% Tween 20 (PBST) for 30 min and then incubated overnight with the primary antibodies in 5% skimmed milk in PBST. Membranes were then washed and incubated with horseradish peroxidase (HRP)-conjugated secondary antibodies for 30 min at room temperature. Secondary antibodies were detected using Immobilon Western Chemiluminescent HRP Substrate (Millipore). The experiment was performed according to the manufacturer’s instructions.

### Quantitative Reverse Transcription PCR

Total RNA was extracted from the human eSCs using TRIzol reagent (Thermo Fisher Scientific, Waltham, MA, United States), and 2 μg of this RNA was used to synthesize cDNA using the TOPscript^TM^ RT DryMIX kit (dT18 plus) following the manufacturer’s instructions (Enzynomics, Daejeon, South Korea). Quantitative reverse transcription PCR (qRT-PCR) was conducted using TOPreal^TM^ qPCR 2X PreMIX (SYBR Green with high ROX) (Enzynomics) and a CFX384 C1000 Thermal Cycler (Bio-Rad, Hercules, CA, United States). Human *L19* was used to normalize *IL-8* expression, and *glyceraldehyde 3-phosphate dehydrogenase* (*GAPDH*) was used to normalize the expression of the other genes. The primers used for qRT-PCR, such as for *FOXO1, GAPDH, IGFBP1, PRL* (Baek et al., 2018), *L19* (Francis et al., 2006), *p16* (Zhang et al., 2017), and *p53* (Zhou et al., 2017) have been previously reported. Primers for *IL8* and *p21* are listed in Supplementary Table 4.

### Cell Proliferation and Viability

The proliferation of human eSCs was evaluated using a cell counting kit (CCK)-8 (Dojindo Laboratories Kumamoto, Japan) following the manufacturer’s instructions. In addition, 5-ethynyl-2′-deoxyuridine (EdU) labeling was performed as previously reported (Son et al., 2019). Briefly, the cells were pulsed with Edu (10 μM) for 2 h before harvesting. Then, the cells were fixed in 3.7% formaldehyde for 15 min and labeled with an Edu mixture containing ascorbic acid, CuSO_4_, and fluorescein (FAM) azide at final concentrations of 500, 100, and 10 mM, respectively, for 30 min at room temperature in the dark. After incubation with 4′,6′-diamidino-2-phenylindole (DAPI) (1:5,000) for 20 min, the cells were observed under a fluorescent microscope (Olympus CKX3-Houn Microscope; Olympus, Tokyo, Japan) equipped with a 20X objective. The fluorescent images were captured using Retiga R6 (Qimaging, Surrey, BC, Canada). EdU-labeled cells were counted using an ImageJ cell counter.

### Analysis of Apoptotic Cells

Apoptosis was examined using a TUNEL assay kit according to the manufacturer’s protocol (Promega, Madison, WI, United States).

### Cell Cycle Analysis

The cells were collected by centrifugation at 1000 × *g* for 5 min at a concentration of 3 × 10^5^ cells per tube and washed twice with PBS. The cell pellets were suspended in 70% ethanol (1 mL) at 4 °C for 1 h and washed again once with PBS. They were then re-suspended in 0.5 mL of propidium iodide (PI, 50 mg/L) and 1.5% RNase A (7 mg/mL) for 30 min at 37 °C in the dark, and analyzed using flow cytometry (BD FACS Calibur; BD Biosciences, San Jose, CA, United States).

### β-Galactosidase Staining

The cells were stained with senescence-associated β-galactosidase (senescence-associated β-galactosidase staining kit, Cell Signaling Technology) according to the manufacturer’s instructions. The cells were photographed using an Imager Z1 (Zeiss) microscope equipped with a 5X objective (Oberkochen, Land Baden-Württemberg, Germany). Senescent cells were detected as blue-stained, and the staining intensity of positive cells was scored as 0 (staining absent), 1 (partial cytoplasmic staining), and 2 (total cytoplasmic staining). A total of 300 cells were counted in three random fields of view for each sample.

### Protein Phosphatase Assay

Protein phosphatase activity was measured as previously reported (McAvoy and Nairn, 2010). Briefly, the cells were lysed using passive lysis buffer (Promega) and reacted with p-nitrophenylphosphate for 45 min in a colorimetric assay buffer (20 mM Tris pH 7.5, 5 mM MgCl_2_, 1 mM ethylene glycol-bis (β-aminoethyl ether)-N,N′,N′,N-tetraacetic acid (EGTA), 0.02% β-mercaptoethanol, and 0.1 mg/mL bovine serum albumin). The absorbance was measured at 405 nm. The lysate containing the phosphatase inhibitor was used as a blank.

### Lentivirus-Mediated Short Hairpin RNA and Transfection

Protein phosphatase catalytic subunit a (PPP1Ca) shRNAs were obtained from Sigma-Aldrich in the pLKO.1-puro vector (MISSION shRNA). Clone IDs were PP1C-1, TRCN0000002452, PP1C-2, and TRC0000002455, and the lentivirus packaging and testing were performed as previously described (Baek et al., 2018).

### Statistical Analysis

All quantitative data are represented as the mean ± standard deviation (SD) of at least three independent experiments. All data points are shown as dots in all quantified graphs. Means were calculated from the results of 3 to 6 independent experiments for all figures. The exact sample size for each experiment is described in the figure legends. Where necessary, the statistical significance of the data was determined using a two-tailed paired Student’s *t*-test using Excel. The specific types of tests and the *P*-values, when applicable, are indicated in the figure legends. *P*-values < 0.05 were considered statistically significant.

## Results

### C-Peptide Decreases Decidualization in Human Endometrial Stromal Cells

**FIGURE 1:**
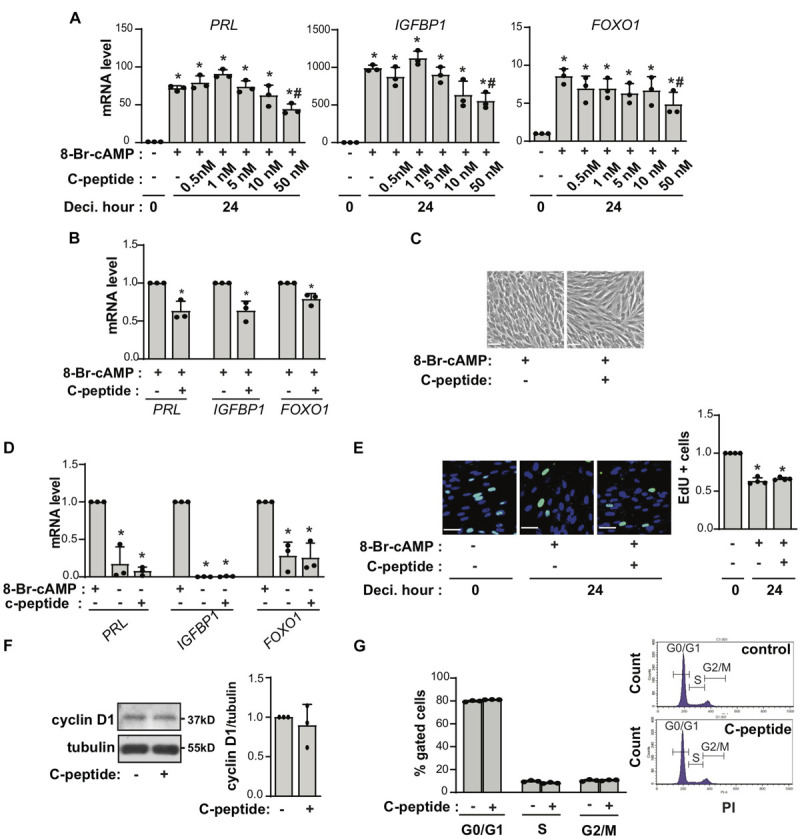
C-peptide decreases decidualization in human endometrial stromal cells. **(A)** Human eSCs were induced to decidualize with 0.5 mM 8-Br-cAMP in the presence of an indicated concentration of C-peptide for 24 h. The cells were then lysed and subjected to qRT-PCR (*n* = 3). **(B)** qRT-PCR results for human eSCs from three different people (*n* = 3) treated with 0.5 mM 8-Br-cAMP with or without 50 nM C-peptide for 24 h. **(C)** Cells were induced to differentiate in the presence of 8-Br-cAMP with or without 50 nM C-peptide for 4 days and photographed using Nikon Eclipse Ti2 microscope (X4). Scale bar = 100 μm (*n* = 3). **(D)** Human eSCs were induced to differentiate in the presence of 0.5 mM 8-Br-cAMP for 2 days; subsequently, the 8-Br-cAMP was withdrawn, and the cells were incubated for further 2 days. The cells were lysed and analyzed using qRT-PCR (*n* = 3). **(E)** Cells were induced to decidualize using 0.5 mM 8-Br-cAMP in the presence or absence of 50 nM C-peptide for 24 h and stained for detecting EdU incorporation. The cells were photographed using Olympus CKX53 microscope (X20). Scale bar = 50 μm (*n* = 3). **(F)** Cells were treated with 50 nM C-peptide for 24 h and then subjected to western blot analysis (*n* = 3). **(G)** Cells were treated as in **(E)**, stained with PI, and analyzed using flow cytometry (*n* = 3). *P < 0.05, **P < 0.01 versus an undifferentiated control; *^#^P* < 0.05 vs. a differentiated control. Human eSCs from two to three different people were used.

To assess the effect of C-peptide on decidualization in human eSCs, the cells were treated with 0.5 mM 8-Br-cAMP in the presence or absence of C-peptide. The cells were differentiated in the presence of 8-Br-cAMP as shown by increased mRNA expression of decidualization markers, including *PRL*, *IGFBP1*, and *FOXO1* (Figure 1A). Notably, treatment with C-peptide significantly decreased the mRNA expression of *PRL*, *IGFBP1*, and *FOXO1* in a concentration-dependent manner (Figure 1A). The low concentration of C-peptide (1 nM) started to decrease *PRL* mRNA expression after induction of decidualization for 2 days (Supplementary Figure 1A), and inhibited for 3 or 6 day-decidualization (Supplementary Figures 1A,B). Primary eSCs from different individuals were differentiated using 8-Br-cAMP induction over a 2 day-period in the presence or absence of 50 nM C-peptide, and the results of their differentiation confirmed the antideciduogenic effect of C-peptide (Figure 1B). In addition, human eSCs remained fibroblast-like in the presence of 8-Br-cAMP and 50 nM C-peptide for 4 days as observed under a microscope, whereas the cells treated with only 8-Br-cAMP were enlarged and round (Figure 1C). The antideciduoginic effect in C-peptide and 8-Br-cAMP-co-treated cells for 4 days were proved by the decrease in mRNA expression of decidualization markers (Supplementary Figure 1C). When 8-Br-cAMP was withdrawn from the decidualized human eSCs, human eSCs underwent dedifferentiation and reverted to fibroblast-like cells with low levels of *PRL* and *IGFBP1*, as previously reported (Yoon et al., 2007; Figure 1D). This reversal in the presence of C-peptide was comparable to the observations made in the absence of C-peptide (Figure 1D; second bar vs. third bar), indicating that C-peptide did not facilitate the restoration of the growth status of human eSCs. In addition, C-peptide did not affect human eSCs exiting the cell cycle, as reflected by no changes in EdU-positive cell staining during c-AMP-induced decidualization (Figure 1E), along with no changes in cyclin D1 expression (Figure 1F), or in the G1, G2, and S phase ratios of PI-stained cells (Figure 1G).

### C-Peptide Increases Cellular Senescence and Decreases Apoptosis During Decidualization

**FIGURE 2:**
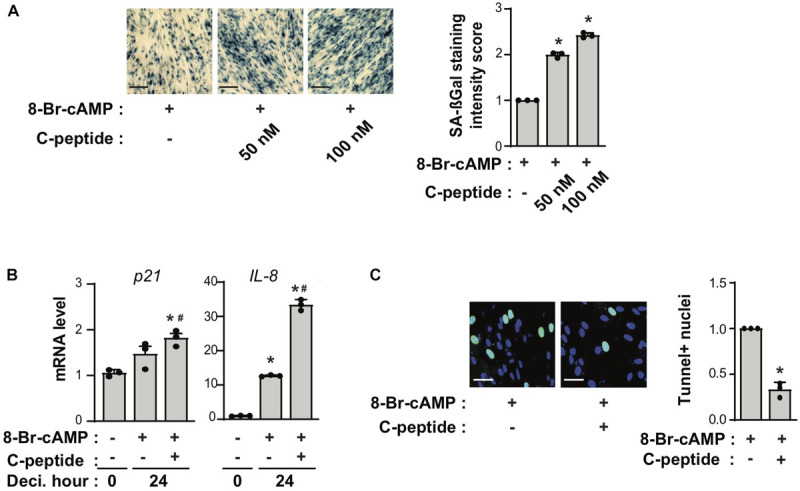
C-peptide increases cellular senescence but decreases apoptosis. **(A)** Human eSCs were induced to differentiate in the presence of 0.5 mM 8-Br-cAMP with or without 50 nM or 100 nM C-peptide for 4 days, and cellular senescence was measured using a senescence-β-galactosidase (SA-βG) assay. The cells were photographed by Zeiss imager Z1 microscope (X5). The staining intensity of the positive cells was scored as 0 (staining absent); 1 (partial cytoplasmic staining); or 2 (total cytoplasmic staining). Scale bar = 100 μm (*n* = 3). **(B)** The cells were decidualized with 0.5 mM 8-Br-cAMP in the presence or absence of 50 nM C-peptide for 24 h. These cells were then lysed and subjected to qRT-PCR analysis (*n* = 3). **(C)** The cells were induced to differentiate in the presence of 0.5 mM 8-Br-cAMP with or without C-peptide for 2 days and then subjected to the TUNEL assay. The cells were photographed using Olympus CKX53 microscope (X20). Scale bar = 50 μm (*n* = 3). *P < 0.05, **P < 0.01 vs. an undifferentiated control; ^#^*P* < 0.05 versus a differentiated control. Human eSCs from two to three different people were used.

Senescence-associated β-galactosidase (SAβG)^+^ cell numbers increase during decidualization, whereas cellular senescence prevents the differentiation of endometrial mesenchymal stem cells in decidualized cultures (Brighton et al., 2017). When cells were decidualized with 8-Br-cAMP in the presence of C-peptide, SAβG^+^ eSCs increased in a dose-dependent manner (Figure 2A). Although this marker for cellular senescence is expressed heterogeneously depending on both the cell type and the insult, the stimulus of senescence increases the expression of cell cycle arrest mediators such as p21 as well as secreted factors of senescence-associated secreted phenotypes (SASP) such as IL-8 (Deryabin et al., 2020). The mRNA levels of senescence markers *p21* and *IL-8* increased significantly 24 h after treatment with 8-Br-cAMP and C-peptide (Figure 2B). In addition, apoptosis was decreased, as demonstrated by a decrease in the number of dead-end TUNEL-positive cells during 8-Br-cAMP-induced decidualization (Figure 2C). Apoptosis increases during c-AMP-induced decidualization (Leno-Duran et al., 2014) and senescence counteracts apoptosis in a p21-dependent manner (Munoz-Espin et al., 2013). Thus, these observations suggest that C-peptide leads to an increase in decidual senescence and a decrease in apoptosis, resulting in defective decidualization.

### C-Peptide Potentiates the Inhibitory Effect of Either Insulin or Palmitic Acid on Decidualization

**FIGURE 3:**
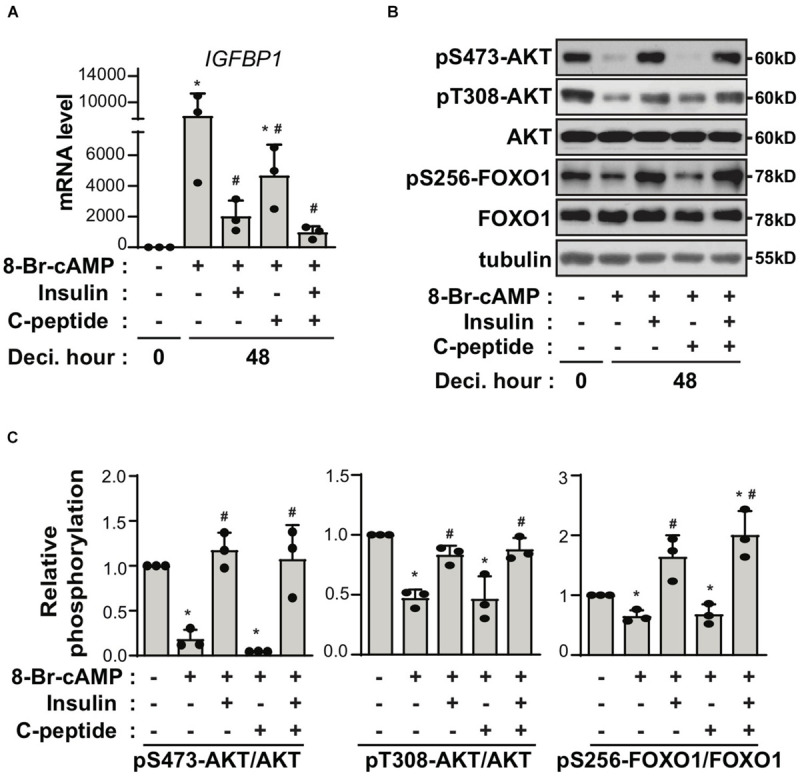
C-peptide inhibits decidualization independent of insulin-induced inhibitory mechanism. **(A)** Human eSCs were induced to differentiate using 0.5 mM 8-Br-cAMP in the presence of 100 nM insulin, 50 nM C-peptide, or cotreatment with insulin and C-peptide for 48 h, followed by cell lysis and qRT-PCR analysis of the RNA samples (*n* = 3). **(B)** The cells were treated as described in **(A)**, followed by lysis, and the lysates were subjected to western blotting (*n* = 3). **(C)** The relative intensities of the bands were quantified using ImageJ analysis software (*n* = 3). Data represent pSer473-AKT and pThr308-AKT as compared to AKT and pSer256-FOXO1 as compared to FOXO1. *P < 0.05 versus an undifferentiated control; ^#^*P* < 0.05 vs. a differentiated control. Human eSCs from two to three different people were used.

**FIGURE 4:**
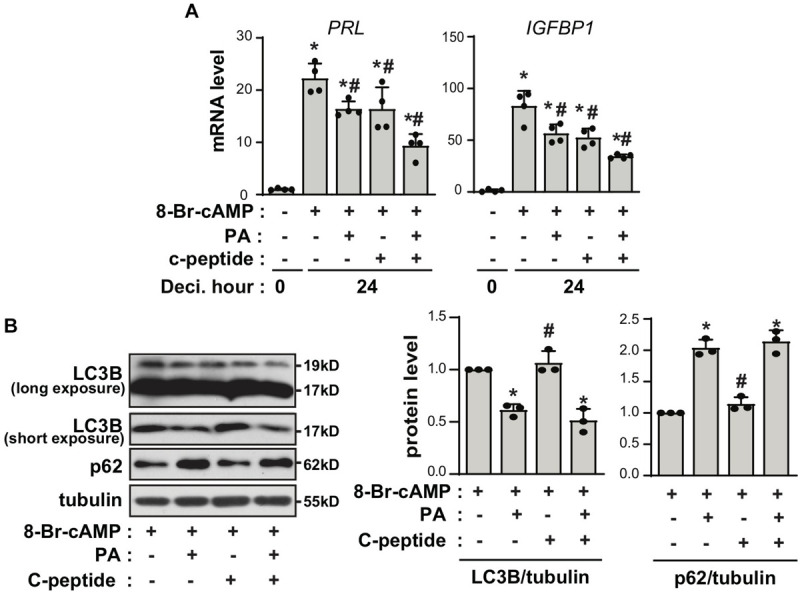
C-peptide potentiates the inhibitory effect of palmitic acid on decidualization through autophagy-independent mechanism. **(A)** Human eSCs were treated with 0.5 mM 8-Br-cAMP in the presence or absence of 100 μM palmitic acid and 50 nM C-peptide for 24 h as indicated. The cells were lysed and subjected to qRT-PCR analysis (*n* = 3). *P < 0.05 vs. an undifferentiated control; ^#^*P* < 0.05 vs. a differentiated control. **(B)** The cells were treated as described in **(A)**, lysed, and the lysates were subjected to western blotting. The relative intensities of the bands were quantified using ImageJ analysis software (*n* = 3). Data represent LC3B as compared to tubulin and p62 as compared to tubulin. *P < 0.05 vs. a 8-Br-cAMP-treated group; ^#^*P* < 0.05 vs. a PA and 8-Br-cAMP-treated group. Human eSCs from two to three different people were used.

C-peptide is produced at equimolar concentrations to that of insulin in pancreatic β-cells during the enzymatic cleavage of proinsulin to insulin (Jones and Hattersley, 2013). Thus, we speculated whether the antideciduogenic effect of C-peptide was regulated by the same pathway as that of insulin. Insulin inhibits decidualization by decreasing the expression of IGFBP1, but not PRL, in a FOXO1-dependent manner (Ujvari et al., 2017). As shown in Figure 3A, treatment with insulin reduced the mRNA expression of *IGFBP1*, as previously reported (Ujvari et al., 2017). Notably, cotreatment with insulin and C-peptide further inhibited *IGFBP1* mRNA expression (Figure 3A), suggesting that C-peptide has an additional inhibitory effect on *IGFBP1* expression in addition to the antideciduogenic effects of insulin. In addition, the phosphorylation of AKT and FOXO1 increased in the presence of insulin during decidualization, as previously reported (Ujvari et al., 2017), whereas they remained unchanged following treatment with C-peptide (Figures 3B,C). Moreover, cotreatment with insulin and C-peptide did not potentiate the phosphorylation of AKT and FOXO1 (Figures 3B,C), indicating that C-peptide inhibited 8-Br-cAMP-induced decidualization independent of AKT/FOXO1 signaling. In addition, C-peptide levels are reportedly increased in obese women with hyperinsulinemia (Poniedziałek-Czajkowska et al., 2018). Palmitic acid (PA) is the most abundant unsaturated fatty acid in the current western diet and contributes to hyperlipidemia (Kien et al., 2014). PA has been reported to inhibit decidualization by decreasing autophagic flux (Rhee et al., 2016). Exposure to 100 μM PA suppressed decidualization, as demonstrated by a decrease in mRNA expression of *PRL* and *IGFBP1*, as previously reported (Rhee et al., 2016), and co-treatment with PA and C-peptide reduced *PRL* and *IGFBP1* mRNA expression (Figure 4A). However, autophagic flux remained unchanged in the presence of C-peptide, whereas it was dampened in the presence of PA or the combination of C-peptide and PA (Figure 4B), suggesting that C-peptide regulates decidualization via a mechanism different from autophagic flux.

### C-Peptide Decreases the Phosphorylation of GSK3β at Ser9 in a PP1-Dependent Manner

**FIGURE 5:**
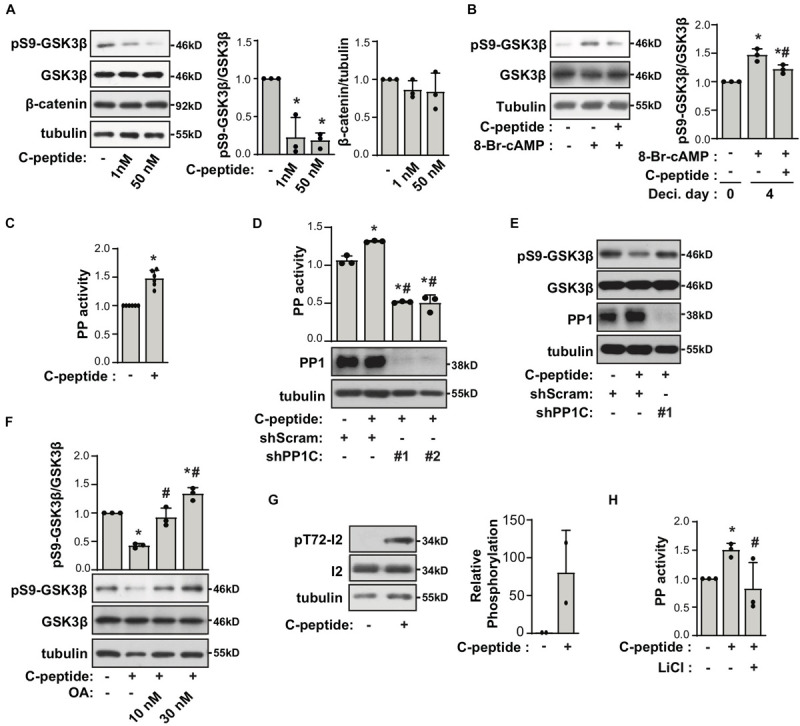
C-peptide decreases the phosphorylation of GSK3β in PP1 activity-dependent manner. **(A)** Human eSCs were serum-starved for 18 h and treated with 1 nM or 50 nM C-peptide for 10 min. The cells were lysed and subjected to western blotting (*n* = 3). **(B)** Cells were treated with 0.5 mM 8-Br-cAMP in the presence of 50 nM C-peptide for 4 days. The cells were lysed and subjected to western blotting (*n* = 3). **(C)** Human eSCs were serum-starved for 18 h and treated with 50 nM C-peptide for 10 min. The cells were lysed and subjected to a protein phosphatase (PP) assay using p-nitrophenyl phosphate and evaluated at 405 nm (*n* = 6). **(D,E)** Human eSCs were transduced with shScram and shPP1C and selected using 2 μM puromycin for 5 days. Cells were serum-starved and treated with 50 nM C-peptide for 10 min. **(D)** PP activity was evaluated as same as **(C)**. **(D,E)** The lysates were analyzed by western blotting. **(F)** After cells were serum-starved for 18 h and pretreated with 10 nM or 30 nM OA for 1 h, cells were treated with 50 nM C-peptide for 10 min. The lysates were subjected to western blot analysis. **(G)** Cells were treated as **(C)**, lysed and subjected to western blotting (*n* = 3). **(H)** The cells were serum-starved for 18 h, pretreated with 40 μM LiCl for 1 h, treated with 50 nM C-peptide for 10 min, and subjected to PP activity assay (*n* = 3). *P < 0.05 vs. control; ^#^*P* < 0.05 vs. a differentiated control **(B)** or C-peptide-treated cells **(D,F,H)**. The relative intensities of the bands were quantified using ImageJ analysis software. Data represent pSer9-GSK3β as compared to GSK3β **(A,B,F)**, β-catenin to tubulin **(A)**, and pThr72-I2 to I2 **(G)**. Human eSCs from two to four different people were used.

**FIGURE 6:**
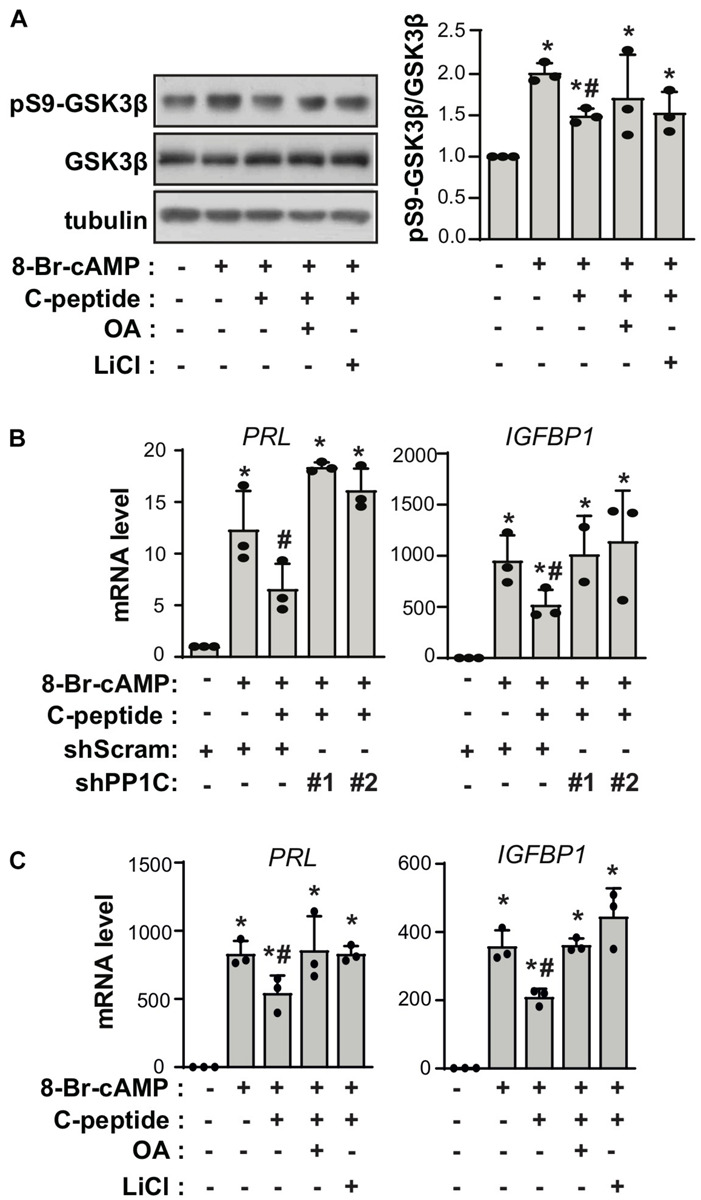
C-peptide inhibits decidualization in a GSK3β-PP1 activity-dependent manner. **(A)** Human eSCs were pretreated with 10 nM OA or 40 μM LiCl for 1 h and induced to differentiate with 0.5 mM 8-Br-cAMP in the presence or absence of C-peptide for 24 h. Cells were then lysed and subjected to western blotting (*n* = 3). The relative intensities of the bands were quantified using ImageJ analysis software. Data represent pSer9-GSK3b as compared to GSK3b. **(B)** The cells were transduced with shScram and shPP1C and selected using 2 μM puromycin for 5 days. The cells were induced to differentiate with 0.5 mM 8-Br-cAMP in the presence or absence of 50 nM C-peptide for 24 h. The cell lysates were subjected to qRT-PCR analysis (*n* = 3). **(C)** The cells were treated as described in **(A)** and subjected to qRT-PCR analysis (*n* = 3). *P < 0.05 vs. an undifferentiated control; ^#^*P* < 0.05 vs. a differentiated control **(A–C)**. Human eSCs from two to four different people were used.

To uncover the mechanism underlying the effect of C-peptide on decidualization, we evaluated the phosphorylation status of GSK3β in C-peptide-treated human eSCs. Inhibition of GSK3β attenuates apoptosis (Souder and Anderson, 2019), and GSK3β blockage is reported to be accompanied by a decrease in the level of SAβG^+^ cells (Zmijewski and Jope, 2004). The changes in GSK3β activity are facilitated by the inhibitory phosphorylation of Ser9 in the amino-terminal motif, which is regulated by several kinases, including AKT, PKA/PKC, and p90RSK (Manning and Toker, 2017). Treatment with C-peptide reduced phosphorylation of GSK3β at Ser9 in undifferentiated human eSCs (Figure 5A) as well as in decidualized eSCs (Figure 5B). The protein level of β-catenin remained unchanged after stimulation with C-peptide (Figure 5A). Notably, C-peptide activated protein phosphatase activity (Figure 5C). Knockdown of PP1 C (catalytic subunit) with two independent shRNAs decreased C-peptide-induced protein phosphatase activity in human eSCs (Figure 5D), suggesting that protein phosphatase activity is primarily facilitated by PP1 activity in C-peptide-stimulated human eSCs. Both PP1C knockdown (Figure 5E) and pretreatment with okadaic acid (OA), an inhibitor of PP1 (Yoon et al., 2005), for 1 h (Figure 5F) restored GSK3β phosphorylation in C-peptide-stimulated cells, implying the involvement of PP1 in the regulation of GSK3β phosphorylation. In addition, C-peptide induced PP1 inhibitor2 (I2) phosphorylation at Thr72 (Figure 5G), which induced the dissociation of I2 from PP1 and subsequent activation of PP1 (Monteserin-Garcia et al., 2013). In line with this result, GSK3β inhibition by LiCl abolished C-peptide-induced protein phosphatase activity (Figure 5H), suggesting that GSK3β activation is required for PP1 protein phosphatase activity via I2 phosphorylation. Additionally, C-peptide-induced reduction in GSK3β phosphorylation was abolished in both OA and LiCl-pretreated human eSCs during decidualization (Figure 6A). mRNA expression of decidualization markers, *PRL* and *IGFBP1*, was restored by PP1C knockdown (Figure 6B), PP1 inhibition by OA, and GSK3β blockage by LiCl (Figure 6C). These results suggest that the antideciduogenic effect of C-peptide is facilitated by a decrease in inhibitory GSK3β phosphorylation, which is achieved using a PP1-dependent mechanism.

### C-Peptide Increases Cellular Senescence and Decreases Apoptosis Using a GSK3β-PP1-Dependent Mechanism

**FIGURE 7:**
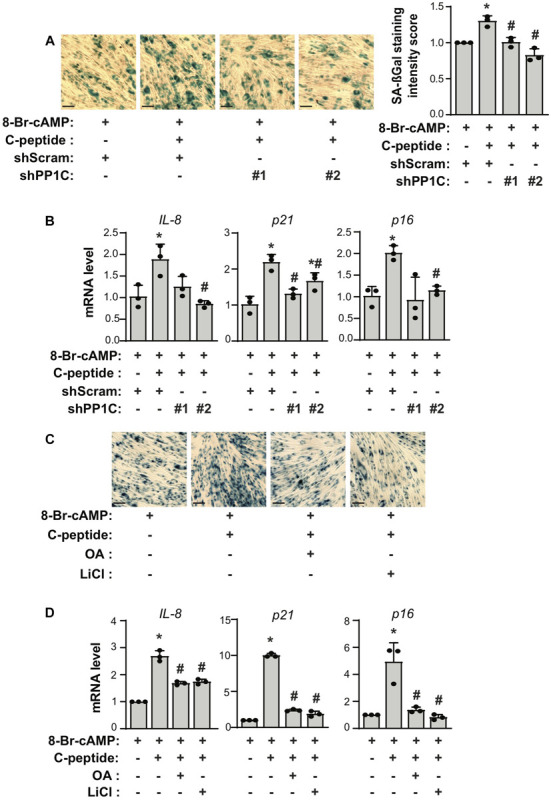
C-peptide increases cellular senescence via PP1-GSK3β. **(A,B)** Human eSCs were transduced with shScram and shPP1C, selected using 2 μM puromycin for 5 days. **(A)** Cells were induced to differentiate with 0.5 mM 8-Br-cAMP in the presence or absence of 50 nM C-peptide for 4 days. Cellular senescence was measured using a senescence-associated β-galactosidase (SA-βG) assay (*n* = 3). **(B)** Cells were differentiated as **(A)** for 24 h. The lysates were subjected to qRT-PCR (*n* = 3). **(C)** Cells were pretreated with 10 nM OA or 40 μM LiCl for 1 h and then induced to differentiate with 0.5 mM 8-Br-cAMP in the presence or absence of 50 nM C-peptide for 4 days. Cellular senescence was measured using SA-βG assay (*n* = 3). **(D)** The cells were treated as described in **(C)** for 24 h, followed by lysis, and the RNA was analyzed by qRT-PCR. *P < 0.05 vs. an undifferentiated control; ^#^*P* < 0.05 vs. a differentiated control. The cells were photographed by imager Z1 Zeiss microscope (X5), Scale bar = 100 μm. Human eSCs from two to four different people were used.

**FIGURE 8:**
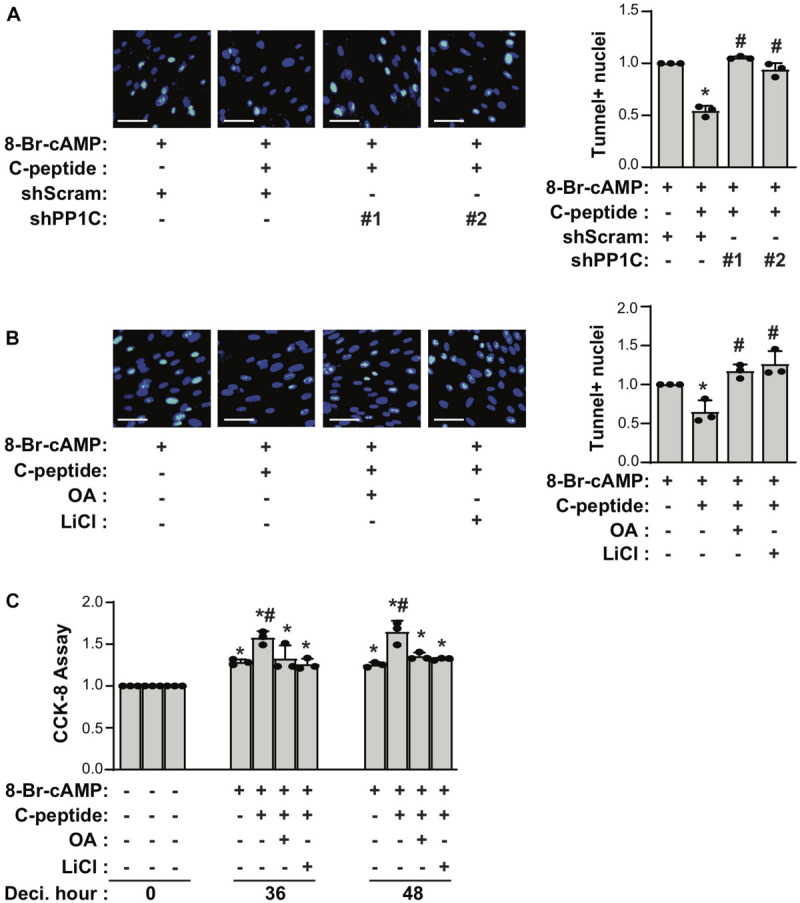
C-peptide inhibits apoptosis in a PP1-GSK3β-dependent manner. **(A)** Human eSCs were transduced with shScram and shPP1C, followed by selection using 2 μM puromycin for 5 days. Cells were induced to differentiate with 0.5 mM 8-Br-cAMP in the presence or absence of 50 nM C-peptide for 2 days. Cells were then subjected to TUNEL assay, and TUNEL+ nuclei were counted using Image J (*n* = 3). **(B)** Cells were pretreated with 10 nM OA or 40 μM LiCl for 1 h and then induced to differentiate with 0.5 mM 8-Br-cAMP in the presence or absence of 50 nM C-peptide for 2 days. Cells were subjected to TUNEL assay (*n* = 3). **(C)** Cells were pretreated with 10 nM OA or 40 μM LiCl for 1 h and then induced to differentiate with 0.5 mM 8-Br-cAMP in the presence or absence of 50 nM C-peptide for the indicated times. The cells were lysed and subjected to CCK-8 assay (*n* = 3). *P < 0.05 vs. an undifferentiated control; ^#^*P* < 0.05 vs. a differentiated control. The cells were photographed using Olympus CKX53 microscope (X20). Scale bar = 50 μm. Human eSCs from two to four different people were used.

If both PP1 and GSK3β are responsible for the antideciduogenic effect of C-peptide in human eSCs, one would expect that PP1 and GSK3β regulated cellular senescence and apoptosis during 8-Br-cAMP-induced decidualization. First, we assessed the effect of PP1C on cellular senescence in human eSCs. The depletion of PP1C using shRNA (Figures 7A,B) or inhibition of PP1 with OA pretreatment (Figures 7C,D) significantly reduced the number of SAβG^+^ eSCs and the mRNA expression of *IL-8*, *p21*, and *p16* during 8-Br-cAMP-induced decidualization. In addition, pretreatment with LiCl significantly decreased SAβG^+^ eSCs and the mRNA expression of *IL-8*, *p21*, and *p16* during 8-Br-cAMP-induced decidualization (Figures 7C,D). The number of TUNEL+ cells in C-peptide-treated PP1C knockdown cells was comparable to that in scramble-infected control cells (Figure 8A). In addition, the number of TUNEL+ cells was also elevated in OA or LiCl-pretreated eSCs in the presence of C-peptide during decidualization (Figure 8B). Consistently, the number of decidualized cells that were pretreated with OA or LiCl remained unchanged as compared to the differentiated control in C-peptide-treated cells, as proved by the CCK-8 assay (Figure 8C). These results suggest that C-peptide plays a vital role in increasing cellular senescence and decreasing apoptosis in a GSK3β- and PP1-dependent manner.

## Discussion

**FIGURE 9:**
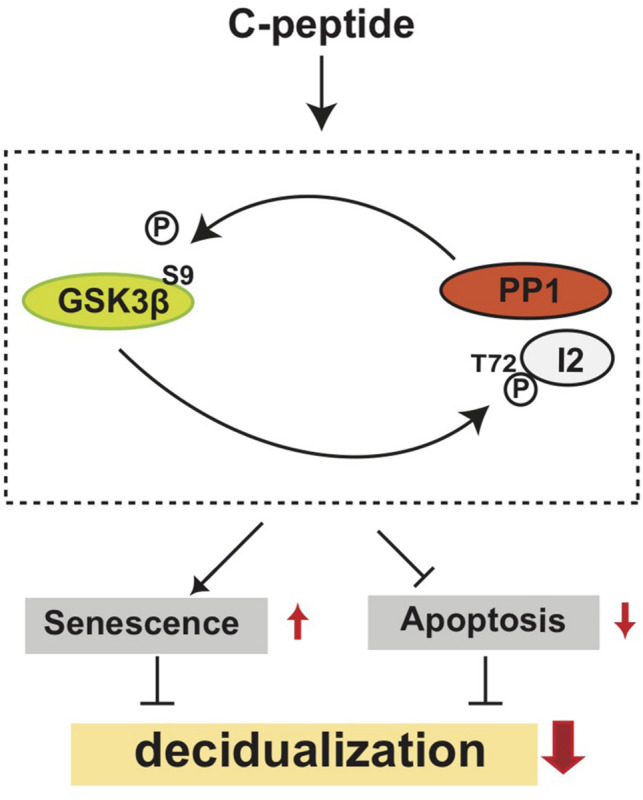
A hypothetical model of the antideciduogenic effect of C-peptide. C-peptide activates PP1, which dephosphorylates GSK3β inhibitory phosphorylation at Ser 9. At the same time, active GSK3β phosphorylates I2 at Thr72, which might result in the dissociation of I2 from PP1. The cross-activation between PP1 and GSK3β increases cellular senescence and decreases apoptosis, leading to the inhibition of decidualization.

C-peptide was initially considered an inert metabolic byproduct, but recently it has been shown to be a critical molecule for the regulation of various cellular and physiological conditions, especially those related to diabetes (Bhatt et al., 2014). This study demonstrates that C-peptide inhibits, but does not completely obstruct decidualization, which increases cellular senescence and decreases apoptosis in human eSCs via a GSK3β- and PP1-dependent mechanism (Figure 9). In addition, C-peptide activated PP1 activity using a GSK3β-dependent mechanism, implying that these proteins create a positive feedback loop. The novel antideciduogenic effect of C-peptide supports the growing belief that C-peptide should be considered a critical factor in embryo implantation and pregnancy maintenance.

The level of C-peptide is normally 0.3–0.6 nM/L under fasting conditions, 1–3 nM/L under postprandial conditions (Leighton et al., 2017), and 0.77–1 nM/L in PCOS patients (Park et al., 2001; Ehrmann et al., 2004; de Medeiros et al., 2018); C-peptide has a half-life of 20–30 min (Jones and Hattersley, 2013). In the present study, we utilized 0.5–100 nM C-peptide; in most cases 50 nM was used. Treatment with physiological levels of C-peptide (1 nM) for 10 min with dephosphorylated GSK3β (Figure 5A), suggested that the physiological concentration of C-peptide is effective enough to induce cellular signaling in human eSCs. In contrast, longer treatment with 1 nM C-peptide (more than 48h) is required to decrease the mRNA expression of *PRL* (Supplementary Figure 1A). Hence, we assumed that a high concentration of C-peptide is required for maintaining the effective level of C-peptide for 24–96 h (1–4 days) following the induction of decidualization. Importantly, to provide continuous supplementation with C-peptide over a long period, it should be administrated in vivo by using subcutaneously implanted osmotic pumps (Bhatt et al., 2013; Lim et al., 2015; Jeon et al., 2019). This ongoing supplementation is indispensable for testing the effect of C-peptide in decidualization in vivo. Along with the inhibitory role of C-peptide in decidualization *in vitro*, further investigation with osmotic pumps is required for determining the function of C-peptide in decidualization *in vivo*.

Identification of the C-peptide receptor is necessary to decipher the mode of action and the biological role of C-peptide in diabetes-related complications. Previous studies have suggested that C-peptide is associated with the activation of a G protein-coupled receptor (GPCR) with GPR146 identified as a potential C-peptide receptor following the investigation of 136 orphan GPCRs using ligand-receptor matching methodologies (Yosten et al., 2013). The knockdown of GPR146 inhibited C-peptide-induced c-Fos expression in the gastric carcinoma cell line KATO III (Yosten et al., 2013). C-peptide was co-localized with GPR146 on the KATO III cell membrane, leading to the internalization of GPR146 (Yosten et al., 2013). However, a recent study did not support the finding that GPR146 is the C-peptide receptor (Lindfors et al., 2020). They utilized dynamic mass distribution, arrestin binding assays with GPR146 overexpressing cells, and cellular binding and internalization under a fluorescent microscope. None of these assays demonstrated reliable binding of GPR146 and C-peptide, or internalization of GPR146 in the presence of C-peptide. Since studies on C-peptide-related receptors in the endometrium have not been reported, further investigations need to be performed to examine the receptors and their C-peptide-induced antideciduogenic effects in the endometrium, possibly focusing on the highly expressed GPRs, including GPR139 or GPR161 (https://www.proteinatlas.org/ENSG00000180269-GPR139/tissue).

In this study, we found that C-peptide activated PP1, leading to dephosphorylation of GSK3β at Ser9 and activating GSK3β in human eSCs. Notably, PP1 was shown to be the primary effector of protein phosphatase activity in human eSCs (Figure 5). Spatial and temporal PP1 activity is regulated by the interactions between PP1 and the phosphatase-interacting proteins (PIP) (Pradhan et al., 2017). In the present study, GSK3β induced I2 phosphorylation at Thr72, which induced the dissociation of I2 from PP1 to activate PP1 activity (Monteserin-Garcia et al., 2013). Both knockdown and inhibition of PP1 restored GSK3β inhibitory phosphorylation, and pretreatment with LiCl also decreased C-peptide-induced protein phosphatase activity. GSK3β, an isoform of GSK, functions as a critical regulator of cell growth and development (Souder and Anderson, 2019). The activity of GSK3β is regulated by its inhibitory phosphorylation and sequestration in the cytosolic complex by Wnt, and GSK3β inactivation promotes cellular proliferation and differentiation by regulating the phosphorylation of cyclin D1 or β-catenin (Rider et al., 2006). Thus, the cross-activation of PP1-GSK3β is essential to the antideciduogenic function of C-peptide, although the primary effector between PP1 and GSK3β needs to be confirmed.

In addition, we found that GSK3β activity is associated with an increase in SAβG^+^ cells and a decrease in apoptotic cells in C-peptide-treated decidual cells. GSK3β has been reported to translocate to the nucleus and interact with p53 in old, senescent WI-38 human fetal lung fibroblasts. In addition, treatment with lithium, a GSK blocker, reduced the levels of p53, p21, and SAβG^+^ WI-38 cells (Zmijewski and Jope, 2004). Furthermore, we observed that C-peptide increased the number of SAβG^+^ eSCs but decreased that of apoptotic eSCs in a GSK3β-PP1-dependent manner. The interaction between cellular senescence and apoptosis depends on the cell type (Childs et al., 2014). Senescent cells affect the growth or apoptotic program of neighboring cells by secreting soluble factors known as SASP (Childs et al., 2014). Conversely, apoptosis resistance could induce cellular senescence; apoptotic stimuli such as UVB or high-dose H_2_O_2_ promote cellular senescence and cell survival by upregulating antiapoptotic Bcl-2 (Childs et al., 2014). Moreover, serum withdrawal leads to replicative cellular senescence together with anti-apoptotic signaling via an increased level of Bcl-2 in human fibroblasts (Childs et al., 2014). Which signal between cellular senescence and apoptosis acts primarily in the stimulation of C-peptide needs to be further investigated.

The link between impaired decidualization of eSCs and the development of senescence has recently been investigated. An increase in p21 expression was accompanied by cellular senescence in both NEDD8 and exchange protein activated by cAMP2 (EPAC2)-calneticulin-inhibited decidualization (Kusama et al., 2014; Liao et al., 2015). In addition, there was a subpopulation of senescent decidual cells, resulting in the presence of various senescent characteristics (p53 stabilization, enhanced expression of CDK inhibitor p16, decreased expression of lamin B1, histone H3 trimethylation, cell size increase, and SA-β-Gal activity). The decidual cells became polarized to form a mature subpopulation of acutely senescent decidual cells, which were abolished by uterine natural killer (uNK) cells in an interleukin 15 (IL-15)-dependent manner (Ochiai et al., 2019). Furthermore, senescent decidual cells have been hypothesized to develop from these presenescent, undifferentiated populations, which exert a significant influence on the secretory profile of the cells during decidualization (Deryabin et al., 2020). Immune cells clear senescent decidual cells, decreasing the number of emerging progesterone-resistant secondary senescent decidual cells, blocking in recurrent pregnancy loss (Lucas et al., 2020). Whether C-peptide regulates the immune cell population, including uNK cells, during decidualization warrants further investigation.

Our study is the first to establish an antideciduogenic role for C-peptide in human eSCs (Figure 9). C-peptide also controls the activation of GSK3β and PP1, thereby modulating cellular senescence and apoptosis during decidualization. This study provides useful insights into the physiological role of C-peptide in hyperinsulinemia conditions and expands our understanding of the underlying mechanism of recurrent pregnancy loss in patients with diabetes and PCOS.

## Data Availability Statement

The original contributions presented in the study are included in the article/Supplementary Material, further inquiries can be directed to the corresponding author/s.

## Ethics Statement

All experiments were performed in compliance with the relevant guidelines and regulations of Gachon University (GAIRB2018-301). The patients/participants provided their written informed consent to participate in this study.

## Author Contributions

SAK and M-SY designed the study. SAK, H-JC, and M-OB performed the experiments. SJC provided human endometrium samples. SAK and M-SY wrote the original draft of the manuscript, and reviewed and edited the manuscript. All authors have read and approved the final manuscript.

## Conflict of Interest

The authors declare that the research was conducted in the absence of any commercial or financial relationships that could be construed as a potential conflict of interest.

## Abbreviations

8-Br-cAMP: 8-bromo adenosine 3′5′-cyclic adenosine monophosphate
Deci: decidualization
FOXO1: Forkhead box protein
IGFBP1: insulin-like growth factor binding protein 1
LiCl: Lithium chloride
I2: protein phosphatase1 inhibitor 2
OA: Okadaic acid
PA: palmitic acid
PP: protein phosphatase
PRL: prolactin
shPP1C: shRNA for PPP1Ca
shScram: shRNA for scramble.
