# Supplementary material for: C-Peptide Inhibits Decidualization in Human Endometrial Stromal Cells via GSK3β-PP1
Source: Front Cell Dev Biol. 2020 Nov 30;8:609551. doi: 10.3389/fcell.2020.609551 (PMC7734312; doi:10.3389/fcell.2020.609551)
Supplement: Supplementary file 1 [file Table_1.docx]

Supplementary Material

**Supplementary Table 1. List of antibodies used in this study**

| **Antibodies** |  | **Cat No** | **Dilution rate (concentration)** | **Company, Location** |
| --- | --- | --- | --- | --- |
| AKT |  | 9272S | 1:1000 (0.034 μg /ml) | Cell Signaling Technology  (Danvers, MA­­,USA) |
| pS473-AKT |  | 4051L | 1:1000 | Cell Signaling Technology  (Danvers, MA,USA) |
| pT308-AKT |  | 9275S | 1:1000 (0.141 μg /ml) | Cell Signaling Technology  (Danvers, MA,USA) |
| β-catenin |  | 9582 | 1:1000 | Cell Signaling Technology  (Danvers, MA,USA) |
| Cyclin D1 |  | SC-450 | 1:200 (1 μg /ml) | Santa cruz biotechnology  (Dallas, TX, USA) |
| Foxo1 |  | 2880S | 1:2000 (0.088 μg /ml) | Cell Signaling Technology  (Danvers, MA,USA) |
| pS256 Foxo1 |  | 9461S | 1:700 (0.107 μg /ml) | Cell Signaling Technology  (Danvers, MA,USA) |
| I2 |  | MAB4719 | 1:200 (2.5 μg /ml) | R&D systems  (Minnesota,USA) |
| T72-I2 |  | ab27850 | 1:200 | abcam, (Cambridge, MA, USA) |
| PP1 |  | MAB3000 | 1:200 (2.5 μg /ml) | R&D systems  (Minnesota,USA) |
| GSK3β |  | 9315 | 1:1000 | Cell Signaling Technology  (Danvers, MA,USA) |
| pS9-GSK3β |  | 9336 | 1:1000 (0.087 μg /ml) | Cell Signaling Technology  (Danvers, MA,USA) |
| LC3B |  | NB100-2220 | 1:5000 (0.2 μg /ml) | Novus Biologicals  (Colorado,USA) |
| P62 |  | 5114S | 1:1000 (0.0181 μg /ml) | Cell Signaling Technology  (Danvers, MA,USA) |
| Tubulin |  | ab11304 | 1:2000 (0.46 μg /ml) | abcam, Cambridge, MA, USA |

***** Concentration of antibodies was available, if it was provided in the datasheet.

**Supplementary Table 2. List of reagents used in this study**

| **Reagents** | **Cat. No** | **Supplier, Location** |
| --- | --- | --- |
| Cell counting kit-8 (cck-8) | PK798 | Dojindo Laboratories (USA) |
| Dead End Fluorometric TUNEL System | G3250 | Promega Corporation (Madison, WI, USA) |
| Insulin Solution (Human) | I9278 | SIGMA-Aldrich (St. Louis, MO, USA) |
| Okadaic Acid | ALX-350-003-C100 | Cell Signaling Technology (Danvers, MA, USA) |
| Palmitic Acid | P5585 | SIGMA-Aldrich (St. Louis, MO, USA) |
| Senescence β-galactosidase staining kit | 9860S | Cell Signaling Technology (Danvers, MA, USA) |
| Vectashield, Antifade Mounting Medium with DAPI | H-1200 | VECTOR laboratories Burlingame (CA USA) |

**Supplementary Table 3. The demographics of sample participants**

| ID | Age  (years) | BMI  (kg/m^2^) | Diabetes | PCOS | HbA1c  (g/dL) | TC  (mg/dL) | Glucose  (mg/dL) | Hysterectomy indication |
| --- | --- | --- | --- | --- | --- | --- | --- | --- |
| 1 | 48 | 21.7 | No | No | 10.5 | 149 | 88 | Myoma & adenomyosis with HMB & dysmenorrhea |
| 2 | 46 | 22.3 | No | No | 14.5 | 215 | 86 | Irresponsive to medication (IUD), Myoma & adenomyosis with lower back pain, IMB |
| 3 | 48 | 25.5 | No | No | 9.7 | 214 | 103 | Myoma with HMB, dysmenorrhea & pelvic pain |
| 4 | 49 | 20.7 | No | No | 9 | 155 | 102 | Irresponsive to medication(IUD) myoma & adenomyosis with HMB & dysmenorrhea |
| 5 | 52 | 22.7 | No | No | 9.2 | 237 | 87 | Irresponsive to medication myoma & adenomyosis with HMB & dysmenorrhea |
| 6 | 49 | 22.6 | No | No | 9.6 | 155 | 103 | Myoma uterus, endometrial polyp |
| 7 | 47 | 18.1 | No | No | 8.6 | 105 | 87 | Myoma with HMB & dysmenorrhea |
| 8 | 47 | 27.5 | No | No | 7.3 | N/A | 97 | Myoma & adenomyosis with HMB & dysmenorrhea |

***Abbreviations:** TC ( total cholesterol)**;** HMB (heavy menstrual bleeding)**;** IUD (intrauterine device); IMB **(**Intermentrual bleeding)
*****Normal range for HbA1c 7.0-15.5 g/dL

**Supplementary Table 4. human Primers used in the qRT-PCR analyses**

| **Target Gene** | **Sequence of Primer** |
| --- | --- |
| IL-8 F | CCAAGGAAAACTGGGTGCAGA |
| IL-8 R | TTCACTGATTCTTGGATACCACAG |
| p21 F | TGGAACTTCGACTTTGTCAC |
| p21 R | CACATGGTCTTCCTCTGCT |

**Supplementary Figure 1. C-peptide increases protein phosphatase activity (related to Fig. 1)**

(A) Human eSCs were induced to decidualize with 0.5 mM 8-Br-cAMP in the presence of an indicated concentration of C-peptide for an indicated time. The cells were then lysed and subjected to qRT-PCR (n=3). (B) Cells were differentiated with 0.5 mM 8-Br-cAMP in the presence of an indicated concentration of C-peptide for 6 days and performed qRT-PCR (n=3). (C) Cells were induced to differentiate in the presence of 8-Br-cAMP with or without 50 nM C-peptide for 4 days and then subjected to qRT-PCR (n=3). **P* < 0.05 versus an undifferentiated control; *#P < 0.05* versus a differentiated control. Human eSCs from three-four different people were used.


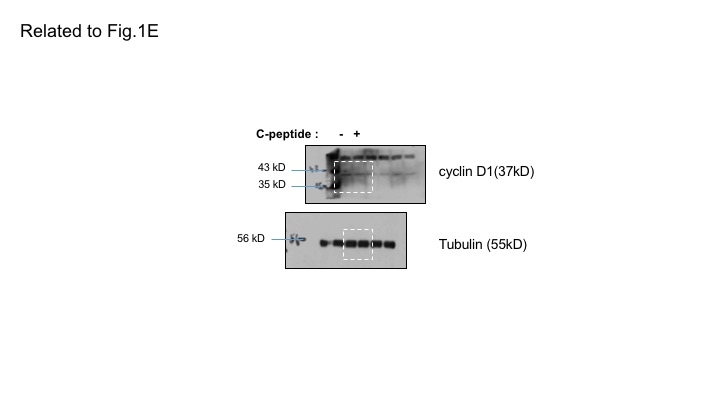


**Supplementary Figure 2. Original blots regarding data shown in Fig. 1F**

The original blots, displayed in Fig. 1F in the main manuscript, are shown here. One batch of blots was chosen to display results representatively. The dotted boxes marked the images in Fig. 1F. The membrane was horizontally cut into pieces, prior to first antibody incubation.


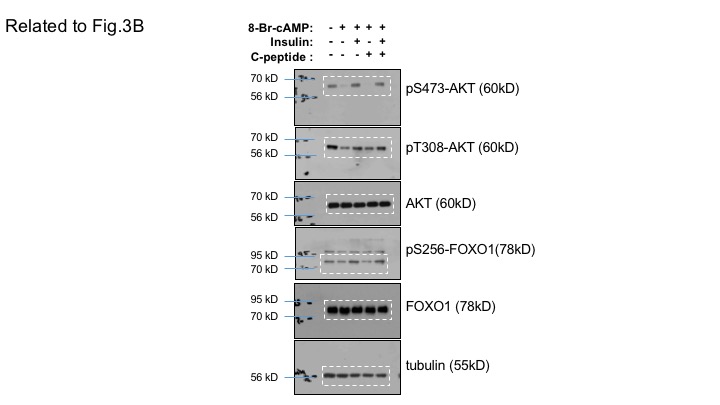


**Supplementary Figure 3. Original blots regarding data shown in Fig. 3B**

The original blots, displayed in Fig. 3B in the main manuscript, are shown here. One batch of blots was chosen to display results representatively. The dotted boxes marked the images in Fig. 3B. The membrane was horizontally cut into pieces, prior to first antibody incubation.


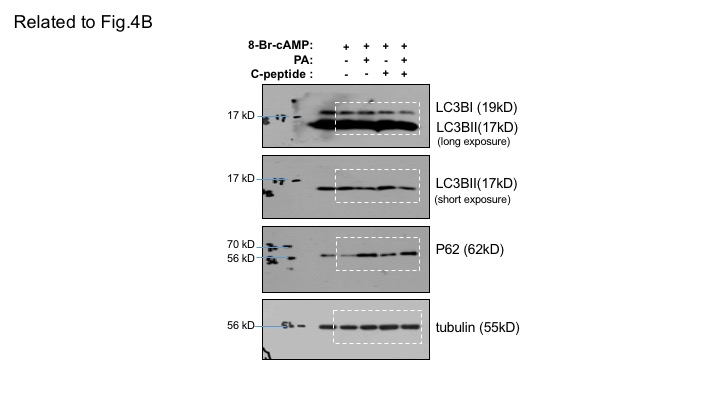


**Supplementary Figure 4. Original blots regarding data shown in Fig. 4B**

The original blots, displayed in Fig. 4B in the main manuscript, are shown here. One batch of blots was chosen to display results representatively. The dotted boxes marked the images in Fig. 4B. The membrane was horizontally cut into pieces, prior to first antibody incubation.


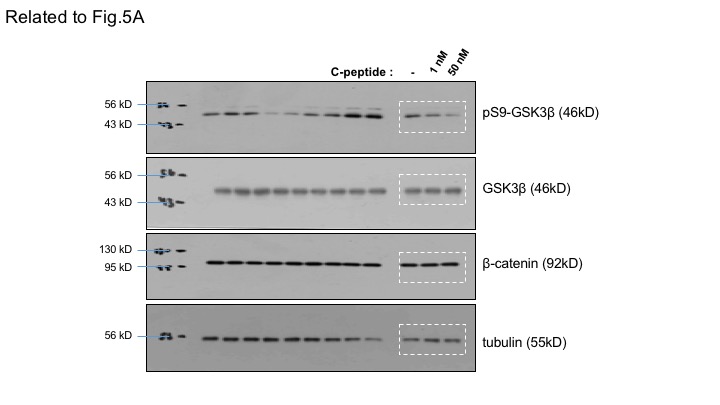


**Supplementary Figure 5. Original blots regarding data shown in Fig. 5A**

The original blots, displayed in Fig. 5A in the main manuscript, are shown here. One batch of blots was chosen to display results representatively. The dotted boxes marked the images in Fig. 5A. The membrane was horizontally cut into pieces, prior to first antibody incubation.

**
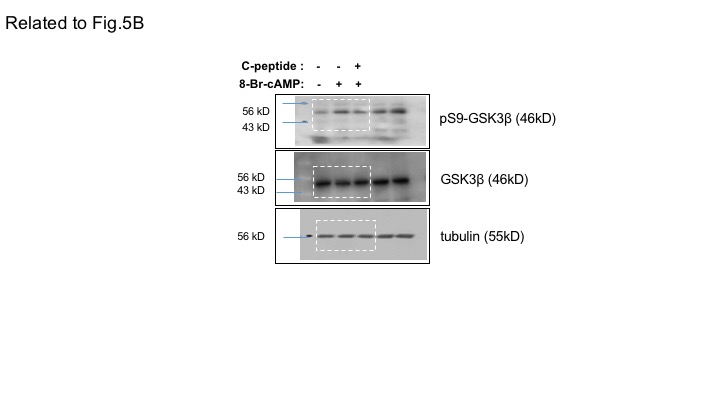
**

**Supplementary Figure 6. Original blots regarding data shown in Fig. 5B**

The original blots, displayed in Fig. 5B in the main manuscript, are shown here. One batch of blots was chosen to display results representatively. The dotted boxes marked the images in Fig. 5B. The membrane was horizontally cut into pieces, prior to first antibody incubation.


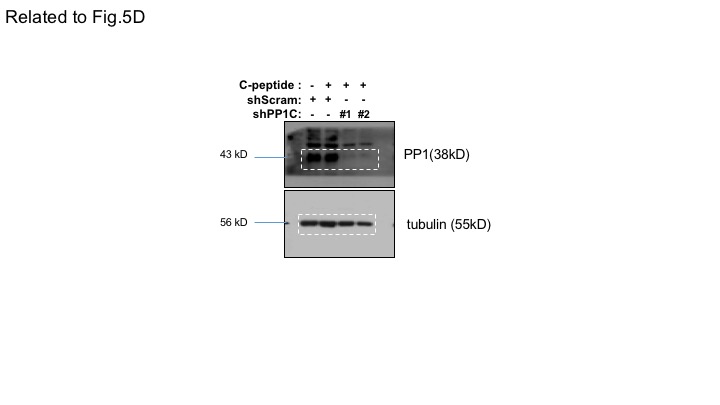


**Supplementary Figure 7. Original blots regarding data shown in Fig. 5D**

The original blots, displayed in Fig .5D in the main manuscript, are shown here. One batch of blots was chosen to display results representatively. The dotted boxes marked the images in Fig. 5D. The membrane was horizontally cut into pieces, prior to first antibody incubation.


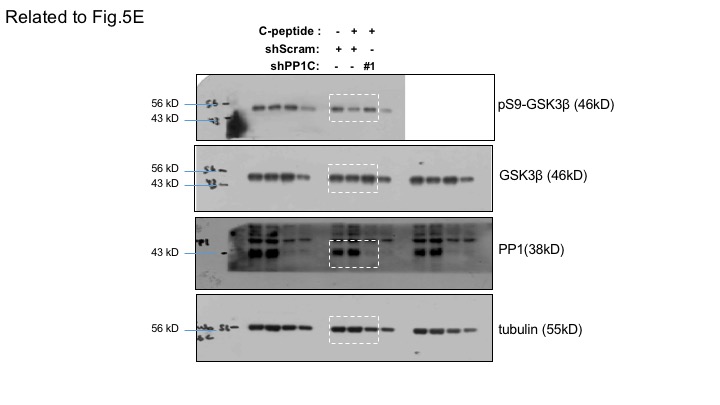


**Supplementary Figure 8. Original blots regarding data shown in Fig. 5E**

The original blots, displayed in Fig. 5E in the main manuscript, are shown here. One batch of blots was chosen to display results representatively. The dotted boxes marked the images in Fig. 5E. The membrane was horizontally cut into pieces, prior to first antibody incubation.

**
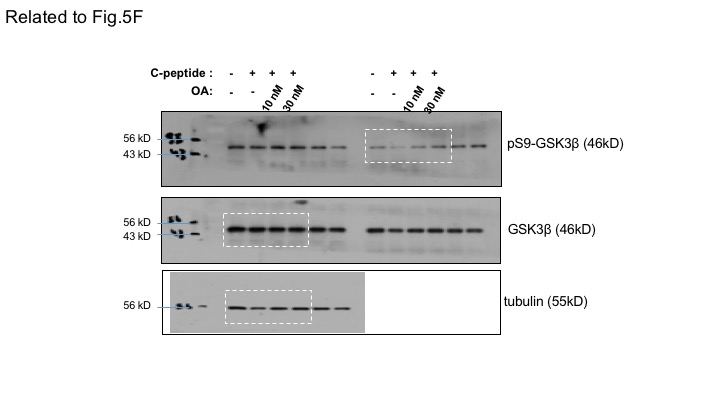
**

**Supplementary Figure 9. Original blots regarding data shown in Fig. 5F**

The original blots, displayed in Fig. 5F in the main manuscript, are shown here. One batch of blots was chosen to display results representatively. The dotted boxes marked the images in Fig. 5F. The membrane was horizontally cut into pieces, prior to first antibody incubation.

**
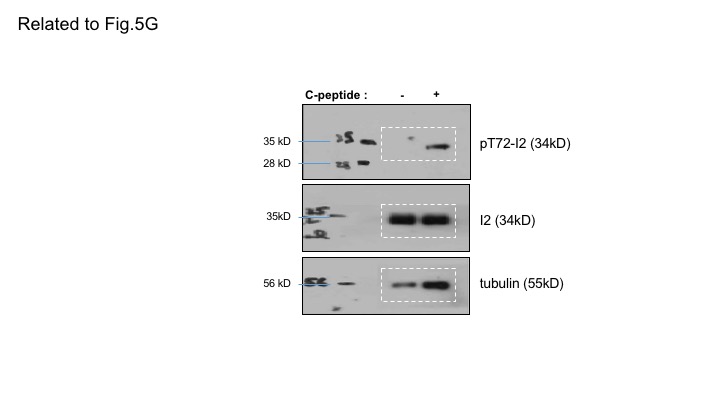
**

**Supplementary Figure 10. Original blots regarding data shown in Fig. 5G**

The original blots, displayed in Fig. 5G in the main manuscript, are shown here. One batch of blots was chosen to display results representatively. The dotted boxes marked the images in Fig. 5G. The membrane was horizontally cut into pieces, prior to first antibody incubation.


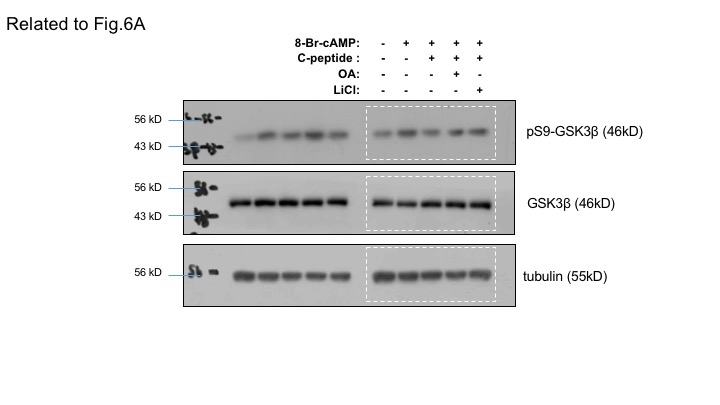


**Supplementary Figure 11. Original blots regarding data shown in Fig. 6A**

The original blots, displayed in Fig. 6A in the main manuscript, are shown here. One batch of blots was chosen to display results representatively. The dotted boxes marked the images in Fig. 6A. The membrane was horizontally cut into pieces, prior to first antibody incubation.
